# EHMTI-0154. Brainstem mechanisms of trigeminal nociception: an fMRI study at 3T

**DOI:** 10.1186/1129-2377-15-S1-E33

**Published:** 2014-09-18

**Authors:** L Schulte, C Sprenger, A May

**Affiliations:** 1 Department of Systems Neuroscience, University Medical Center Hamburg-Eppendorf, Hamburg, Germany

## Introduction

The brainstem is the major site of trigeminal pain processing and modulation and plays a key role in the pathophysiology of various headache disorders. However, comprehensive human imaging studies on function and activity of brainstem areas following trigeminal nociceptive stimulation are scarce.

## Aim

To develope a viable protocol for brainstem fMRI of standardized trigeminal nociceptive stimulation.

## Methods

21 healthy participants (16 female) were scanned on a 3T scanner with a standardized trigeminal nociceptive stimulation protocol for event-related fMRI using a specifically designed sequence for high resolution brainstem echo planar imaging as well as a brainstem specific noise correction technique and brainstem template.

## Results

We observed significant BOLD responses in areas typically involved in trigeminal nociceptive processing such as the spinal trigeminal nuclei (sTN), thalamus, SII, insular cortex and cerebellum as well as in a pain modulating network including the dorsal raphe nuclei (DRN), periaqueductal grey area (PAG), hypothalamus (HT) and nucleus cuneiformis (CN) (p < 0.0002, voxel extent = 10). Using PPI analyses, we found enhanced connectivity of the sTN with the HT and the CN.

## Conclusions

Our results are in line with previous animal and human imaging studies on brainstem processing of nociceptive stimuli. However, using the proposed high resolution imaging technique, we achieved a more detailed insight into brainstem pain processing as compared to whole brain fMRI. High resolution brainstem fMRI of trigeminal nociceptive stimulation offers a unique opportunity to better understand headache pathophysiology.

No conflict of interest.

